# An Unusual Case of ST Elevation Myocardial Infarction in Angiographically Non-Obstructed Coronary Arteries

**DOI:** 10.7759/cureus.12657

**Published:** 2021-01-12

**Authors:** Anup Solsi, Daisy D Gandhi

**Affiliations:** 1 Internal Medicine, Creighton University School of Medicine, St. Joseph's Hospital and Medical Center, Phoenix, USA; 2 Internal Medicine, International American University College of Medicine, Phoenix, USA

**Keywords:** st-elevation myocardial infarction (stemi), myocardial infarction with non-obstructive coronary arteries (minoca), coronary artery angiography

## Abstract

It is routinely believed that patients with electrocardiographic findings of ST elevation myocardial infarction (STEMI) will have evidence of obstructive coronary artery disease on coronary angiography. Although this is the situation in the majority of STEMI instances, in a small percentage of cases, patients presenting with STEMI are found to have minimal to no coronary artery blockage on subsequent angiography, termed MI with non-obstructive coronary arteries (MINOCA). MINOCA is a heterogenous entity with multiple causes, making a focused diagnostic workup important to select an appropriate treatment, with the goal to prevent a mortality similar to obstructive coronary disease. In this case, we describe a unique presentation of MINOCA, after a patient was diagnosed with STEMI.

## Introduction

ST elevation myocardial infarction (STEMI) is classically seen as the consequence of intraluminal plaque rupture leading to transient or persistent total occlusion of the coronary arteries. When STEMI is seen on electrocardiogram (ECG), current American College of Cardiology/American Heart Association practice guidelines recommended early coronary angiography, based on previous groundbreaking studies that showed an obstructive coronary artery in >90% of patients with STEMI [1]. It was from this observation that treatment with percutaneous coronary intervention was developed to improve outcomes in these patients [2].

With the pervasive use of early coronary angiography as the gold standard in myocardial infarction management, multiple studies have reported ~10% of myocardial infarction (MI) patients (including non-STEMI) with no angiographic evidence of obstructive coronary disease (CAD) [3]. This phenomenon has been termed MI with non-obstructive coronary arteries (MINOCA), categorized as angiographically normal or minimal obstructive coronary arteries (less than or equal to 50%) in any potential infarct-related artery [4,5]. MINOCA is reported to be more common in non-STEMI cases compared to STEMI ones, with incidences of 8-10% and 2.8-4.4%, respectively [6]. Due to the scarcity of literature regarding STEMI MINOCA, much of the diagnostic workup and appropriate management remains unknown.

In this report, we present an interesting case of a patient who underwent coronary angiography for an inferior wall STEMI, subsequently found to have lateral/high lateral STEMI with repeat angiography showing no infarct-related coronary disease.

## Case presentation

A 59-year-old Caucasian male with medical history significant for hypertension, hyperlipidemia, deep venous thrombosis, interstitial lung disease, and tobacco dependence for many years presented to the emergency department via emergency medical services with chest pain. The patient reported onset of progressively worsening, mid-sternal chest pain five days ago while at rest, with radiation down bilateral arms. He stated exertion aggravated the pain, while rest alleviated it. He reported positional dizziness, but denied associated diaphoresis, palpitations, nausea, or vomiting. The patient mentioned he had recently been noncompliant with his medications during this time, which included apixaban, atorvastatin, prednisone, losartan, and nifedipine. In the field, the patient was given aspirin 325 mg and sublingual nitroglycerin.

**Figure 1:**
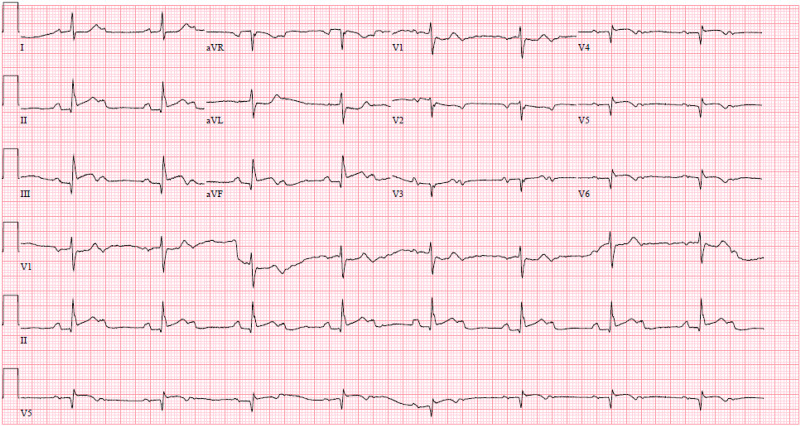
ECG showing Sinus Bradycardia at rate 50 bpm with 2nd degree AV block (Mobitz II), 2:1 conduction, inferior STEMI, reciprocal ST depression in I, aVL. AV - atrioventricular

On arrival, he was noted to be hypotensive with blood pressure 94/54 mmHg and bradycardic at 50 beats per minute (bpm). Examination revealed a diaphoretic, ill-appearing gentleman, with bradycardia, bibasilar inspiratory rales, and diffuse erythematous macules on his skin. There was no jugular venous distension, murmurs, or lower extremity edema. Basic laboratory investigations revealed a leukocytosis of 12.9 x10^3 microliters, sodium 133 mmol/L, potassium 4.6 mmol/L, magnesium 1.9 mg/L and creatinine 2.01 mg/dL (baseline 1.8 mg/dL). Urine drug screen was positive for benzodiazepines and opiates. Initial troponin I was elevated at 20.120 ng/mL. Electrocardiography (ECG) was performed as shown in Figure 1.

**Figure 2:**
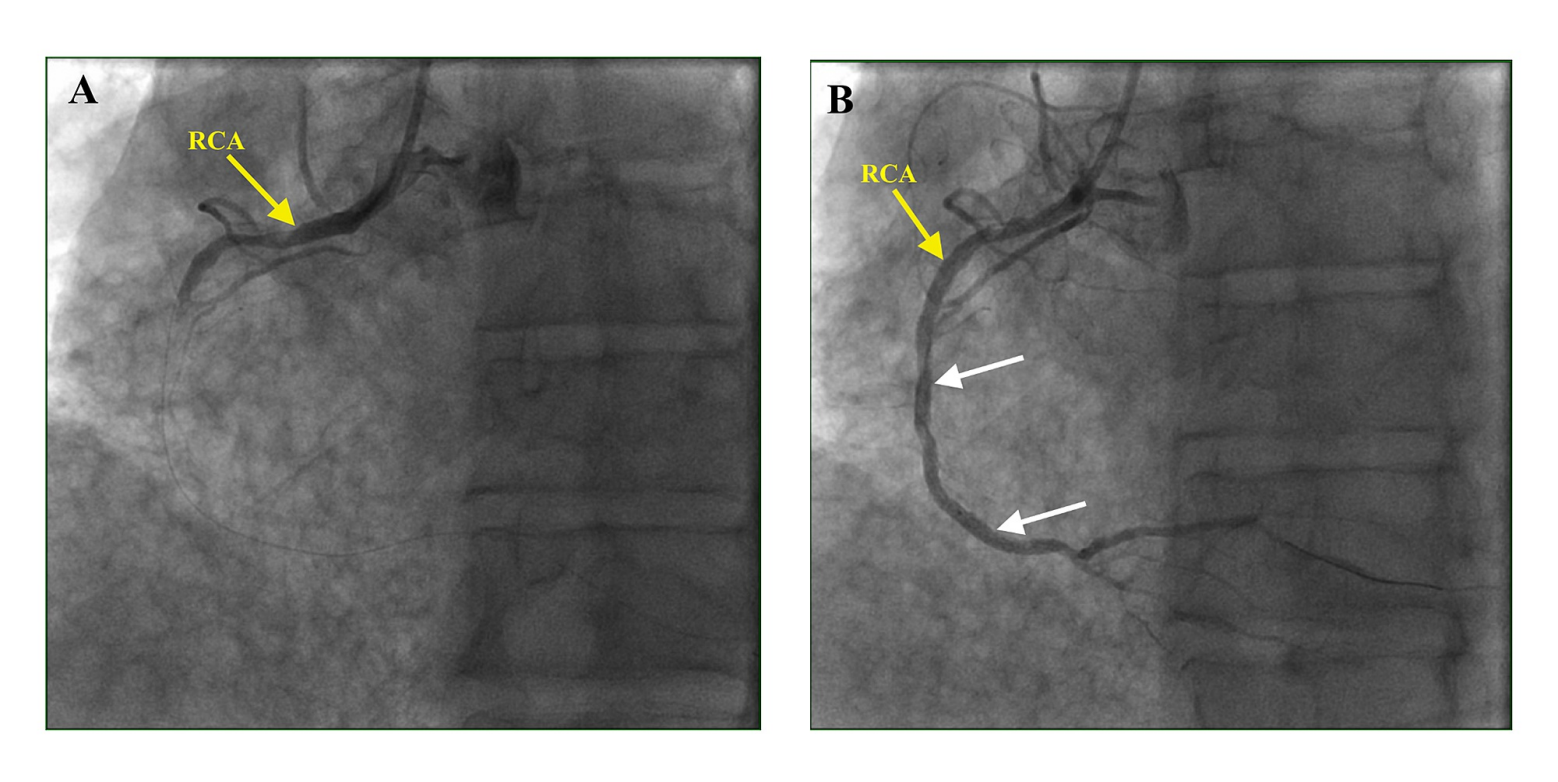
Coronary angiogram. A. Complete occlusion of RCA. B. Post-deployment of DES x2 to mid-proximal and mid-distal segments of RCA, with good angiographic result. RCA - Right Coronary Artery, DES - Drug Eluting Stent

The patient was given additional aspirin 325 mg, atorvastatin 80 mg, and a 1-liter bolus of intravenous fluids. A loading dose of ticagrelor 180 mg was given and a heparin infusion was initiated. Emergently, the patient went for cardiac catheterization. Left heart catheterization demonstrated right coronary artery (RCA) dominant circulation with 100% RCA occlusion (Video 1), treated with two drug-eluting stents (DES) (Figure 2). Angiogram revealed a mild plaque in the mid-left anterior descending (LAD) artery, but no left main (LM) or left circumflex (LCx) disease and left ventricular ejection fraction (LVEF) of 50-55% on left ventriculogram. Coronary angiography of the left system is shown in Video 2, 3.

**Video 1:** Coronary angiogram showing complete occlusion of RCA RCA - Right Coronary Artery

**Video 2:** Coronary Angiography of Left Coronary System

**Video 3:** Coronary Angiography of Left Coronary System

**Figure 3:**
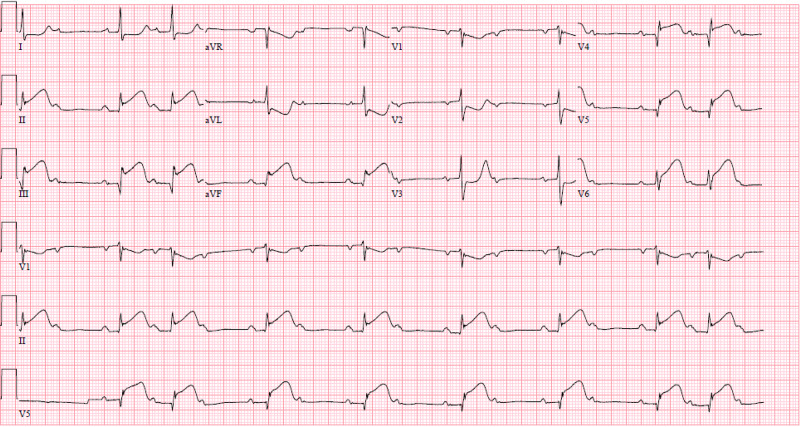
ECG with persistent inferior lead ST elevation, 2:1 Mobitz II AV block, now with new ST elevation in V4-V6.

Post-catheterization, the patient was continued on maintenance dose ticagrelor 90 mg and placed on an eptifibatide infusion for a planned duration of 18 hours (longer durations shown no additional benefit). The patient continued to have chest and bilateral arm pain. He remained hypotensive and bradycardic, with heart rate (HR) in the 40s. Multiple discussions were had regarding temporary cutaneous then venous pacing, however, was not pursued (for unclear reasons). As a result, the patient was initiated on a low-dose dopamine infusion, with improved hemodynamics. Troponin I at this time had trended down to 18.440. Repeat ECG was done and is shown in Figure 3.

**Figure 4:**
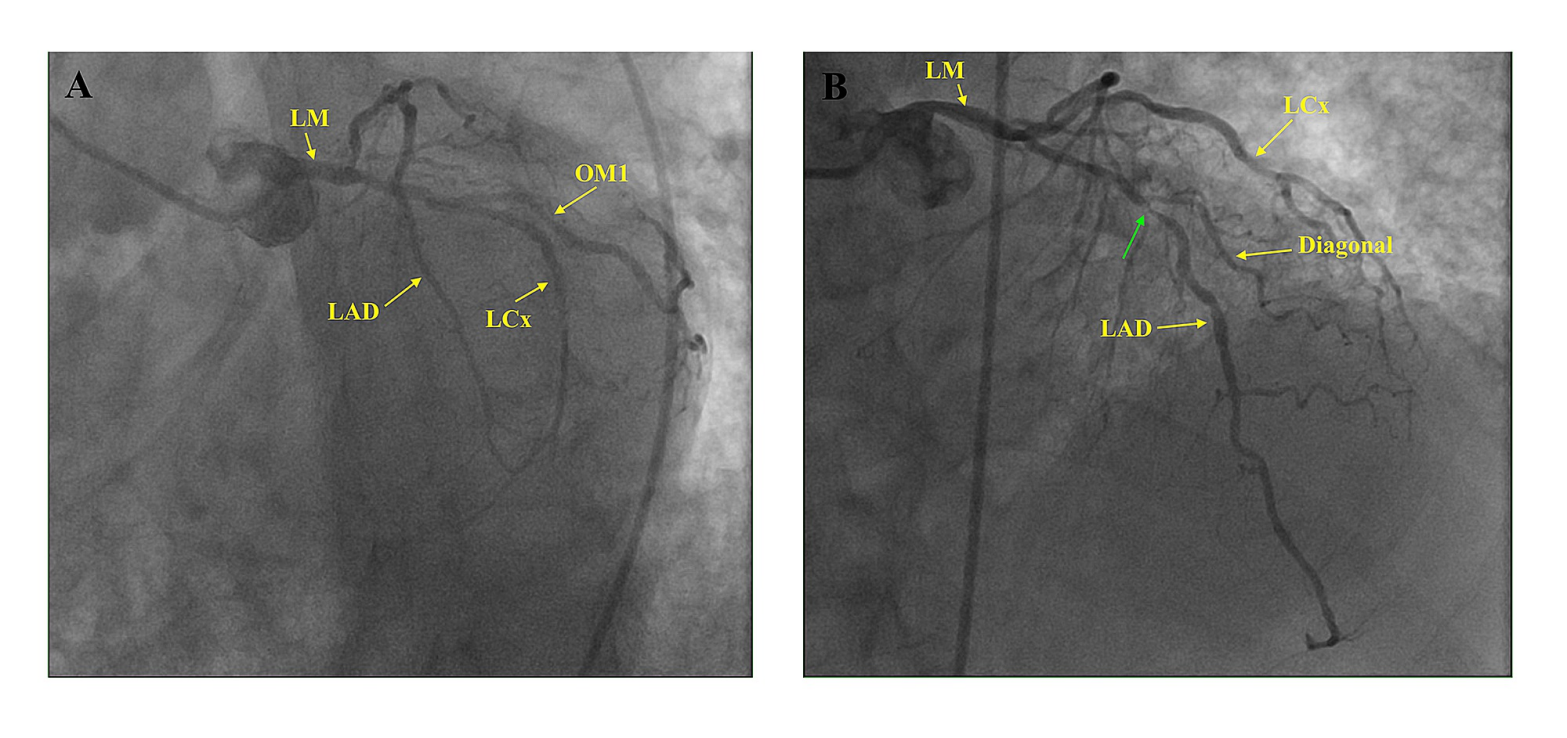
Coronary angiogram. A. LAO caudal view showing patent LM, LAD, LCx and OM1. B. LAO cranial view showing patent LM, LCx, Diagonal branch. There is mild stenosis of mid LAD, distal to first diagonal branch (green arrow). LM – Left main coronary artery, LAD – Left Anterior Descending Artery, LCx – Left Circumflex Artery, OM1 – Obtuse Marginal 1 Artery, LAO – Left Anterior Oblique view.

Based on the repeat ECG with now new lateral ST elevation and persistent inferior STEMI and high degree AV block, the patient was emergently taken back to the cath lab for repeat angiography. Pre-cath, the eptifibatide infusion was stopped, but the dopamine was continued. Angiography revealed approximately 50% stenosis in the LAD and mild disease in the LM or LCx (Videos 4, 5, Figure 4). No intervention was performed. Intravascular ultrasound (IVUS) was not done.

**Video 4:** Repeat Coronary Angiography of Left Coronary System

**Video 5:** Repeat Coronary Angiography of Left Coronary System

Repeat angiography of the RCA showed widely patent stents, no evidence of distal thrombus embolization or coronary vasospasm (Video 6).

**Video 6:** Repeat Coronary Angiography of RCA showing patent stents. There was no throbus or vasospasm seen.

**Figure 5:**
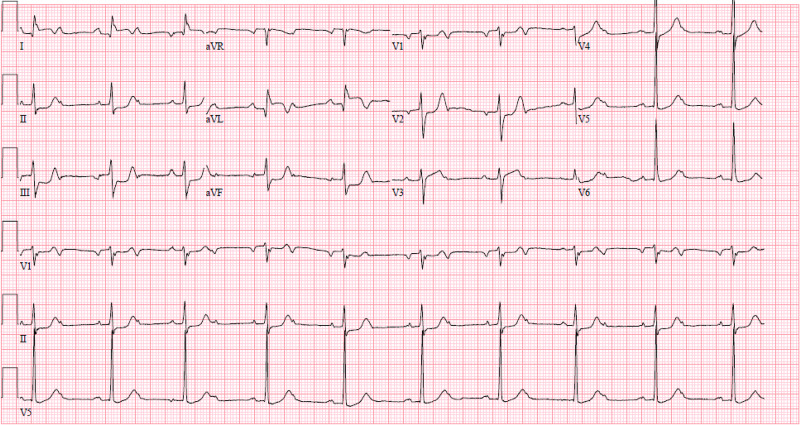
ECG showing sinus bradycardia with 2:1 AV block, lateral wall acute MI/STEMI with ST elevation in leads I, aVL, ST depression inferior leads, V2.

Post-catheterization, the patient remained hypotensive and bradycardic, so norepinephrine infusion was added in addition to the dopamine infusion. With downtrending creatinine to 1.67 mg/dL, normal liver function tests and international normalized ratio (INR), there was minimal concern for developing cardiogenic shock. Transthoracic echocardiogram revealed EF 59%, with hypokinesis of the mid and basal inferior walls of the LV, moderate MR and TR, normal RV size and function. Repeat ECG the following day showed persistent sinus bradycardia with 2:1 AV block with now new high-lateral ST segment elevation (Figure 5). A third angiogram was not performed.

On hospital day two, the patient underwent dual chamber permanent pacemaker (PPM) implantation, as it was believed patient would be unable to tolerate long-term beta-blocker in setting of continued bradycardia. On hospital day four, the patient was discharged in medically stable condition. His hemodynamic status had improved following PPM implantation. As his blood pressure was now stable, low-dose metoprolol 50 mg once daily was added to his medication regimen, along with other goal-directed management therapies, including aspirin 81 mg, atorvastatin 80 mg, lisinopril 2.5 mg, clopidogrel 75 mg, and isosorbide mononitrate 30 mg.

## Discussion

Based on angiography, our patient had no evidence of any disease in what the suspected infarct-related arteries would be with ECG changes suggestive of lateral/high lateral wall STEMI, however did have some disease in the LAD. ST elevation in V4-V6 without ST elevation in V1-V3 is most likely due to LCx or distal diagonal branch occlusion, rather than the main LAD artery [7]. In addition, ST elevation in leads I and aVL with corresponding ST depression in V2 signifies occlusion of the first marginal branch of the LCx [8]. Although ST elevation in leads I and aVL may indicate anterior wall myocardial infarction due to proximal LAD occlusion, ST changes in V2, particularly ST depression, make the LCx and/or its first marginal branch more likely to be the culprit lesion. No ECG changes with ST elevation in leads I, aVL, and subsequently V4-V6 observed in our patient match a mid-LAD stenosis, thus signifying likely MINOCA in our patient. Post-infarction pericarditis is a consideration, however in the absence of diffuse ST segment elevation and the short duration post-infarction, this seemed unlikely.

As it is paramount to discern between MI from obstructive CAD and MINOCA, much research has been done trying to understand and characterize susceptible patients. From comparing various studies, it appears MINOCA is more common in the younger population, with mean age 58.5, compared to those with MI from CAD, seen to have mean age 61.2 [9]. An over-representation of women has been shown in some studies, with others documenting only 40% of MINOCA patients were women [9]. When it comes to cardiovascular risk factors, besides having lower prevalence of hyperlipidemia, patients with MINOCA had similar rates of hypertension, diabetes mellitus, and tobacco use [9].

Proposed causes include unstable coronary plaque, coronary vasospasm, coronary dissection, myocarditis, Takotsubo cardiomyopathy, and even thrombophilic states (Factor 5 Leiden, Protein C/S deficiency) [4]. As the patient had no history of blood clots, these labs were not unfortunately not obtained. Interestingly, atherosclerotic coronary artery plaque rupture/erosion, seen as the major precipitant of STEMI, is not an unusual cause of MINOCA, estimated to be seen in close to one-third of patients presenting with STEMI [10]. Plaque disruption has been observed in patients with near-patent coronary arteries on angiography, thought to be due to coronary artery lumen irregularities [11-13]. Our patient had no angiographic evidence of significant stenosis in the LCx, its obtuse marginal branches or branches of the LAD that would typically be seen in a patient with lateral/high lateral wall MI. It is possible he had some degree of coronary lumen irregularities or a transient occlusion, however, no identification of these changes on two separate angiograms makes this highly unlikely. Furthermore, the patient did not receive any thrombolytics and only had angiographic intervention (balloon dilation) done to the RCA, which would not distort the coronary circulation of the LCx and eliminate any potential obstruction causing the repeat ECG changes. To help identify plaque disruption in MINOCA, the use of intravascular ultrasound and optical coherence tomography has been studied, with reports showing up to 37% of patients with plaque rupture to have non-obstructed coronary arteries [11].

What is more likely is that our patient had clean infarct-related coronary arteries and the no apparent obstruction in the absence of atherosclerosis seen on angiography makes plaque rupture improbable, based on evidence from prior reports [11-13]. As previously mentioned, there was evidence of some non-obstructive disease in his LAD, however this is most likely not the cause of the lateral/high lateral wall STEMI changes on the repeat ECGs. Coronary vasospasm, characterized as a temporary complete or near-complete (>90%) blockage that occurs spontaneously or in response to a provocative stimulus, could be implicated [14]. The stress of active infarction in another coronary artery distribution may have led to a transient LCx or branch occlusion resulting in the ECG changes. Repeat ECG changes showing resolution of the lateral wall STEMI may further prove the vasospasm theory. Testing with provocative spasm using acetylcholine or ergonovine is done to confirm the diagnosis [14]. This was not performed in our patient. Use of Cardiac MRI continues to be explored with MINOCA, with a recent prospective study showing Cardiac MRI was able to identify the etiology of MINOCA in 87% of cases [15].

Data from large MI trials have shown a prevalence of MINOCA to be 1-4% when restricting the MINOCA definition to completely normal coronary arteries (0%), which is much less than the ~10% of patients when using a stenosis cutoff of less than or equal to 50% [16]. This indicates the rarity of MINOCA in patients with angiographically normal coronary arteries, as in our patient. Although our patient met MI criteria with ECG showing STEMI, it is important to consider other modalities to diagnose MI prior to angiography that can still classify as MINOCA. Myocardial perfusion imaging showing new loss of viable myocardium and myocardial functional imaging (ex. echocardiography) depicting new regional wall motion abnormalities followed by non-obstructed coronary arteries on angiography may also be seen in MINOCA [17].

Currently, no guidelines exist to outline the appropriate management strategy for patients with MINOCA. The SWEDEHEART study advocates for the use of beta-blockers, statins, angiotensin-converting enzyme inhibitors/angiotensin receptor blockers, regardless of the MINOCA etiology as these medications have shown long-term benefit, but not P2Y12 inhibitors or dual antiplatelet therapy (DAPT), as these seem to confer no prognostic benefit [18]. We think this is the case due to the increased risk of bleeding with DAPT and with no percutaneous coronary intervention (PCI) usually done for non-obstructive CAD, no true indication exists for DAPT in this population.

The prognosis of MINOCA remains uncertain, with previous studies offering differing statistics. Some studies have shown lower one-year mortality rates compared with MI from obstructive CAD (4.7% vs 6.7%), however, others have found higher one-year mortality (4.7% vs 3.6%) in MINOCA compared with NSTEMI and MI from CAD and increased risk for recurrent MI [4]. Other data report an all-cause mortality rate of 1.1-2.6% at one month and 3.3-6.4% at one year [19].

## Conclusions

MINOCA remains a diverse entity with multiple possible etiologies, often making the clinical evaluation and treatment difficult. We support routine cardiac testing and imaging, including cardiac MRI, to investigate causes for MINOCA. Once coronary angiography is negative, it is imperative to perform a robust diagnostic workup to identify the associated etiology so appropriate management can be initiated. Based on mortality predictions, it is reasonable to treat MINOCA like obstructive CAD until another cause is found.
